# A spatial feature analysis of primary health care utilization in a large city in China and its implications for family doctor contract service policy

**DOI:** 10.1186/s12913-023-10389-8

**Published:** 2024-02-13

**Authors:** Xin Rao, Li Luo, Xingyue Wang

**Affiliations:** 1 Department of General Practice office of General Practice Medical Center, West China HospitalSCU, Chengdu, China; 2 Institute of Hospital Management, West China HospitalSCU, Chengdu, China; 3 Institute of Service ManagementSchool of business, SCU, Chengdu, China; 4 Department of Graduate Medical Education, West China Hospital/School of Medicine, SCU, Chengdu, China

**Keywords:** Spatial feature analysis, Primary health care, Health utilization, Family doctor contract service

## Abstract

**Background:**

Family doctor contract policy is now run by the State Council as an important move to promote the hierarchical medical system. Whether the family doctor contract policy achieves the initial government’s goal should be measured further from the perspective of patient visits between hospitals and community health centers, which are regarded as grass medical agencies.

**Methods:**

The spatial feature measurement method is applied with ArcGIS 10.2 software to analyze the spatial aggregation effect of patient visits to hospitals or community health centers among 20 districts of one large city in China and analyze the family doctor contract policy published in those areas to compare the influence of visit tendencies.

**Results:**

From year 2016-2020, visits to hospitals were in the high-high cluster, and the density was spatially overflow, while there was no such tendency in visits to community health centers. The analysis of different family doctor contract policy implementation times in 20 districts reflects that the family doctor contract policy has a very limited effect on the promotion of the hierarchical medical system, and the innovation of the family doctor contract policy needs to be considered.

**Conclusions:**

A brief summary and potential implications.

A multi-integrated medical system along with family doctor contract policy needs to be established, especially integrated in leadership and governance, financing, workforce, and service delivery between hospitals and community health centers, to promote the hierarchical medical system.

**Supplementary Information:**

The online version contains supplementary material available at 10.1186/s12913-023-10389-8.

## Introduction

Family doctor contract policy [1] is now run by the State Council as an important move to promote the hierarchical medical system [2].

China committed to establishing a CHC-based referral system in a medical reform in 2009. This reform aimed to provide essential medical and public health services for the population by strengthening the community-based system and positioning community health centers (CHCs) [3] as the foundation of and entry point to the health system [4]. However, due to the fragment between the hospital and the CHC [5] in China and the relative weakness [6, 7] of the capability of the CHC [8], medical accessibility is not as ideal as expected [1].

Controlling access to specialty care and moving the focus to managing health by comprehensive and continuous services was expected to reduce health care expenses. Hence, the family doctor contract policy is held by the government with the goal of arranging visits to hospitals and CHCs [9] and promoting the spatial layout of residents’ visits [10–14]. However, due to the free choice for citizens to choose hospitals and CHCs and the relatively week for primary care in China, whether the family doctor contract policy achieves the initial government’s hope, the outcome of government scores and residents’ willingness should be measured further [15].

**Fig. 1:**
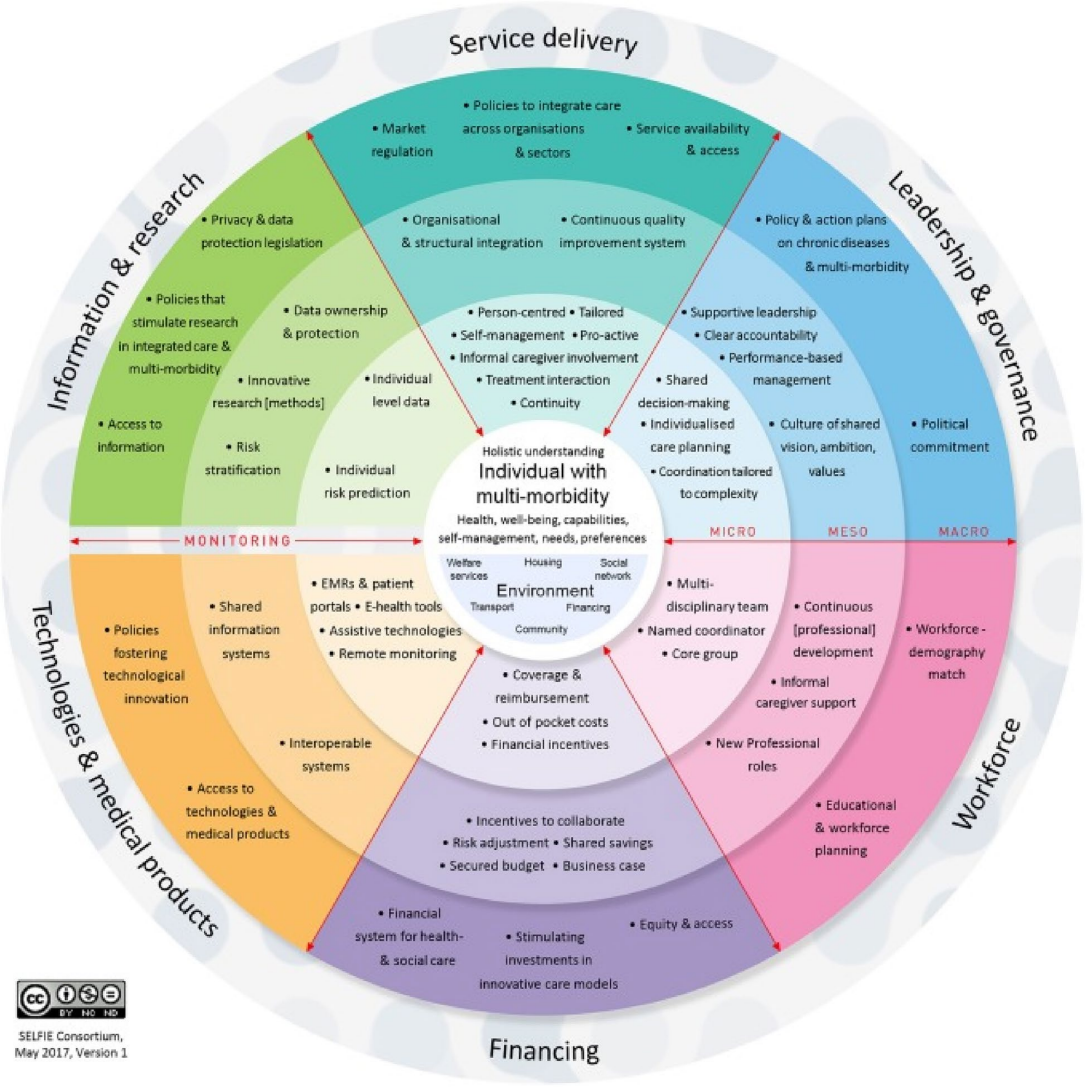
Policy synergy chart

In regard to policy research, it is necessary to consider the synergy of policy impact, including leadership and governance, financing, workforce, service delivery, information and research and technology and medical products (Fig. 1) [16].

Because information&research and techonogies&medical products are mainly criteria for the level of application promotion, the two are not discussed in this study. This study focuses on the policy criteria of leadership and governance, financing, workforce, and service delivery.

The family doctor contract policy for recent years in China is shown in Table 1.

**Table 1:** Family doctor contract policy for recent years in China

Year	Documents	Main content
2011 (http://www.gov.cn/zwgk/2011-07/07/content_1901099.htm)	Guiding Opinions of the State Council on Establishing General Practitioner System, State Council [2011] No.23	General practitioners establish contractual relationship with residents
2016 (http://www.gov.cn/xinwen/2016-06/06/content_5079984.htm)	Guiding Opinions on Promoting Family Doctors' Contract Service (State Council [2016] No.1)	Formally establish a family doctor signing system
2018 (http://www.nhc.gov.cn/cms-search/xxgk/getManuscriptXxgk.htm?id=a3dfc6bfa9774c27bdc86b2a0383467d)	Notice on Doing a Good Job in Family Doctor Signing Service in 2018 (National Health Commission [2018] No.209) Document	Be practical and meticulous to improve the connotation of contracted services
2022 (http://www.gov.cn/xinwen/2022-03/15/content_5679188.htm)	Guiding Opinions on Promoting the High-quality Development of Family Doctors' Contract Services, State Council No.10 [2022]	A number of reform measures are taken simultaneously

The policy document <Guiding Opinions of the State Council on Establishing General Practitioner System> referred to general practitioners establishing contractual relationships with residents in 2011, but it is not a mandatory policy. Some cites are on the frontier of taking the family doctor contract with residents. The policy document <Guiding Opinions on Promoting Family Doctors' Contract Service> was introduced in 2016. In contrast to the Guiding Opinions of the State Council on Establishing a General Practitioner System, it is mandatory because it would be put into the performance item pool of the local government outcome. The performance some reflect the medical staff’s effort but not the citizen’s preference. The policy document < promotion on Family Doctor Contract Service > had been pushed in 2018 because by 2016’s policy, some districts purse the ratio of signing of Family Doctors' Contract, so the need to improve the connotation of contracted services is called. The policy document <Guiding Opinions on Promoting the High-quality Development of Family Doctors' Contract Services> has been run, and a number of reform measures are taken simultaneously, such as the flexibility of the contract subject, contract object, contract content and payment.

In the past, the CHC system faced major challenges, such as a lack of human resources (primary health workers) and a lack of effective gatekeeping mechanisms. In 2011, the function development system was established. In 2016, the Chinese central government issued another important document announcing the establishment of an orderly, efficient, coordinated, and well-functioning referral system. The blueprint for the system conceptualizes FDs as attracting residents to the CFC for their first health care contact, after which critically ill residents are referred to specialists in secondary and tertiary hospitals. Despite these efforts, most patients continue their previous medical-seeking behavior; that is, regardless of the severity of their condition, they flow into major hospitals and visit specialists. On the one hand, disorderly medical treatment will cause crowded medical treatment, which is not conducive to the prevention and control of nosocomial infection; on the other hand, it will lead to waste of medical and health resources. In 2016, the State Office of Health Reform released a document outlining a package of FD contracting services to make it more attractive for residents to contract with an FD and seek first contact at CHC. These contracted services vary by region, city and hospital and offer a range of benefits if residents sign up with FDs. Despite the much effort, the real visit reflects the utilization of the hospital and the primary care. This paper explored the effect of FDs on residents' health care visiting behavior over time by studying visit spatial characteristics to provide empirical evidence for policy makers.

## Methods

### Sample

Visits of the population on average in hospitals in Chengdu city, China, from 2015-2020, including 20 districts in Chengdu city, and visits of the population on average in CHCs in Chengdu city [17].

### Measures and statistical analyses

The spatial measurement method was applied, and the visualize trends, spatial autocorrelation, and high-low density were used in ArcGIS 10.2 software.

Policy document and element capture were applied by web-captuer technology, such as Python software.

## Result

### Primary care spatial features

**Fig. 2:**
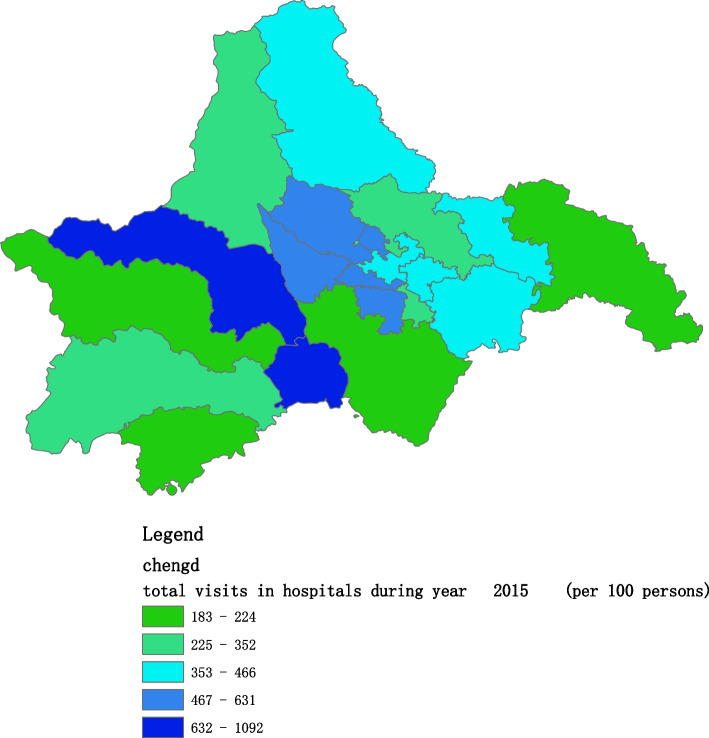
Spatial distribution of residents' visits to primary hospitals in 2015

**Fig. 3:**
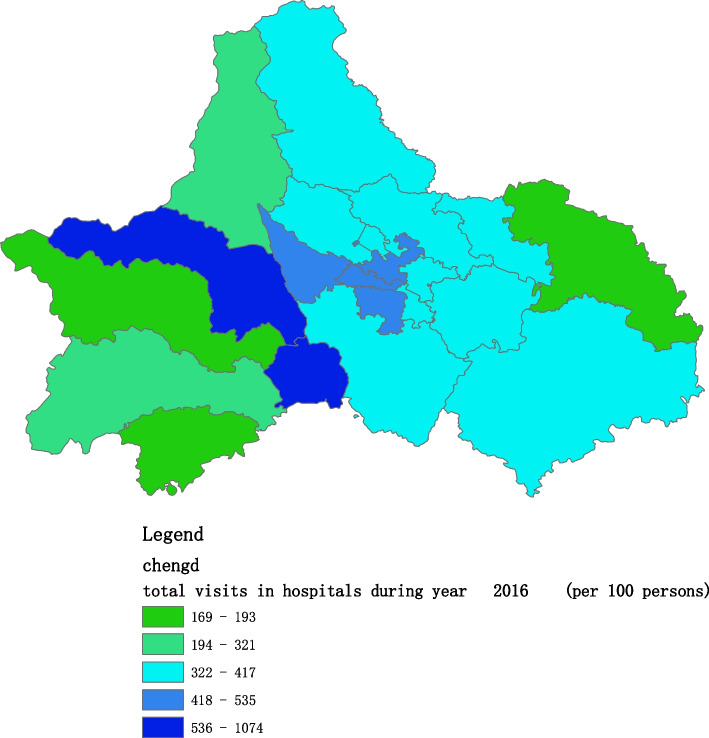
Spatial distribution of residents' visits to primary hospitals in 2016

**Fig. 4:**
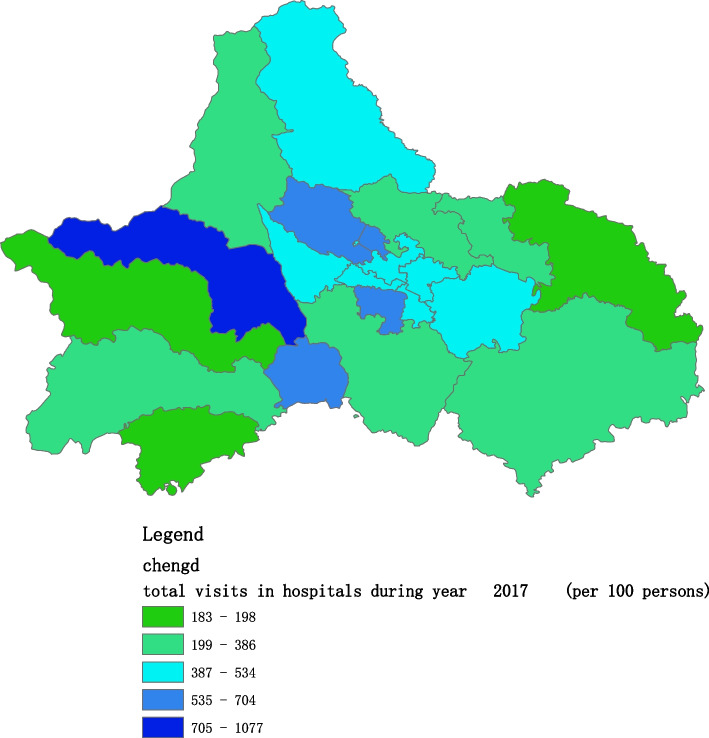
Spatial distribution of residents' visits to primary hospitals in 2017

**Fig. 5:**
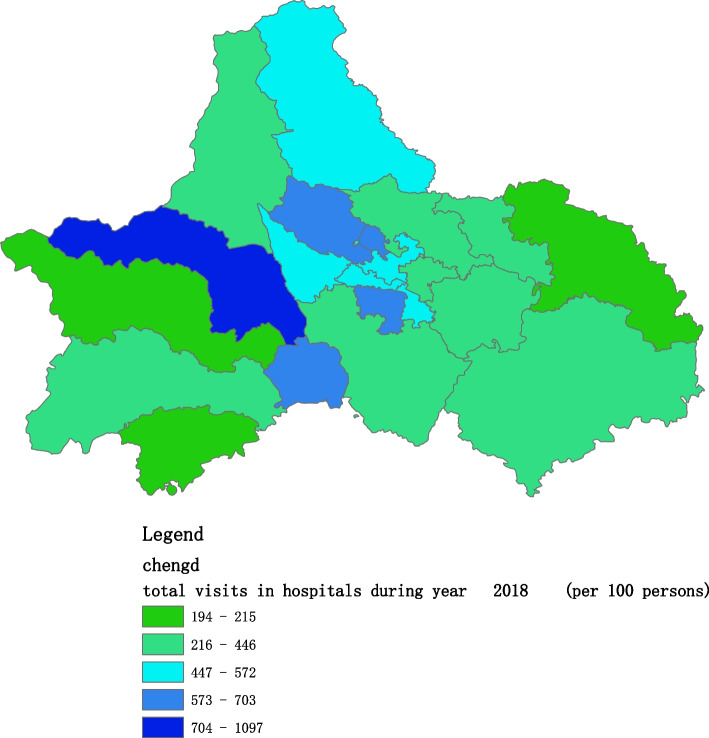
Spatial distribution of residents' visits to primary hospitals in 2018

**Fig. 6:**
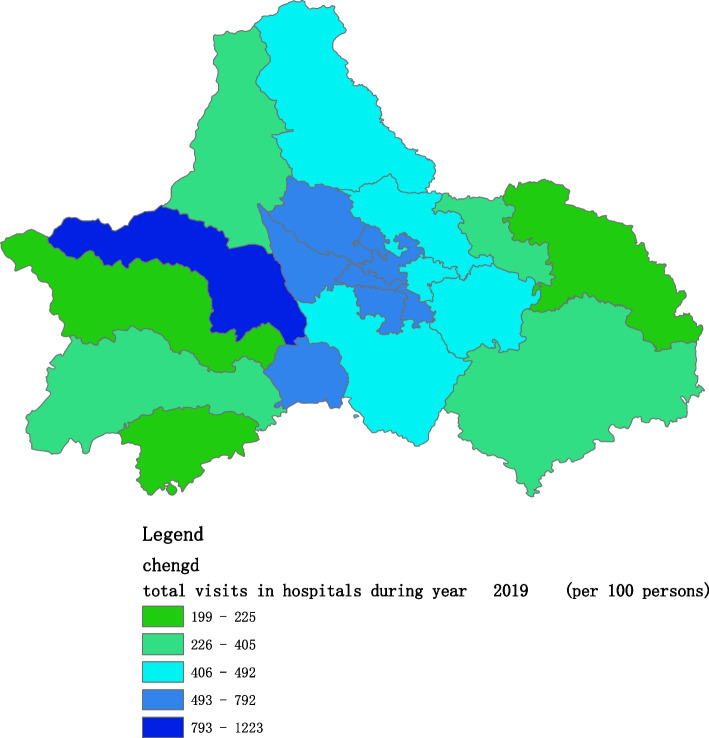
Spatial distribution of residents' visits to primary hospitals in 2019

**Fig. 7:**
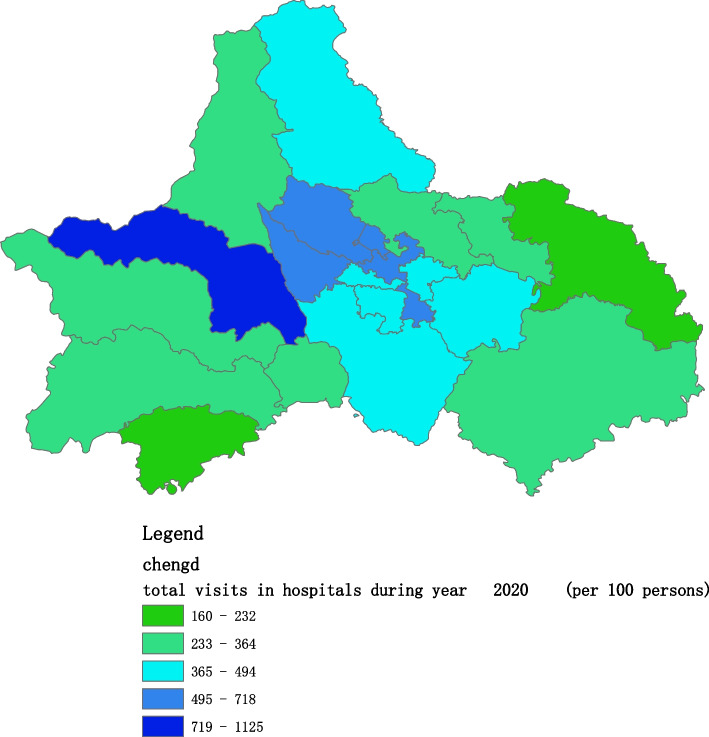
Spatial distribution of residents' visits to primary hospitals in 2020

Figures 2, 3, 4, 5, 6 and 7 below show the average visit of the population in CHC and hospitals in 2015-2020, and different colors show different visit densities.

**Fig. 8:**
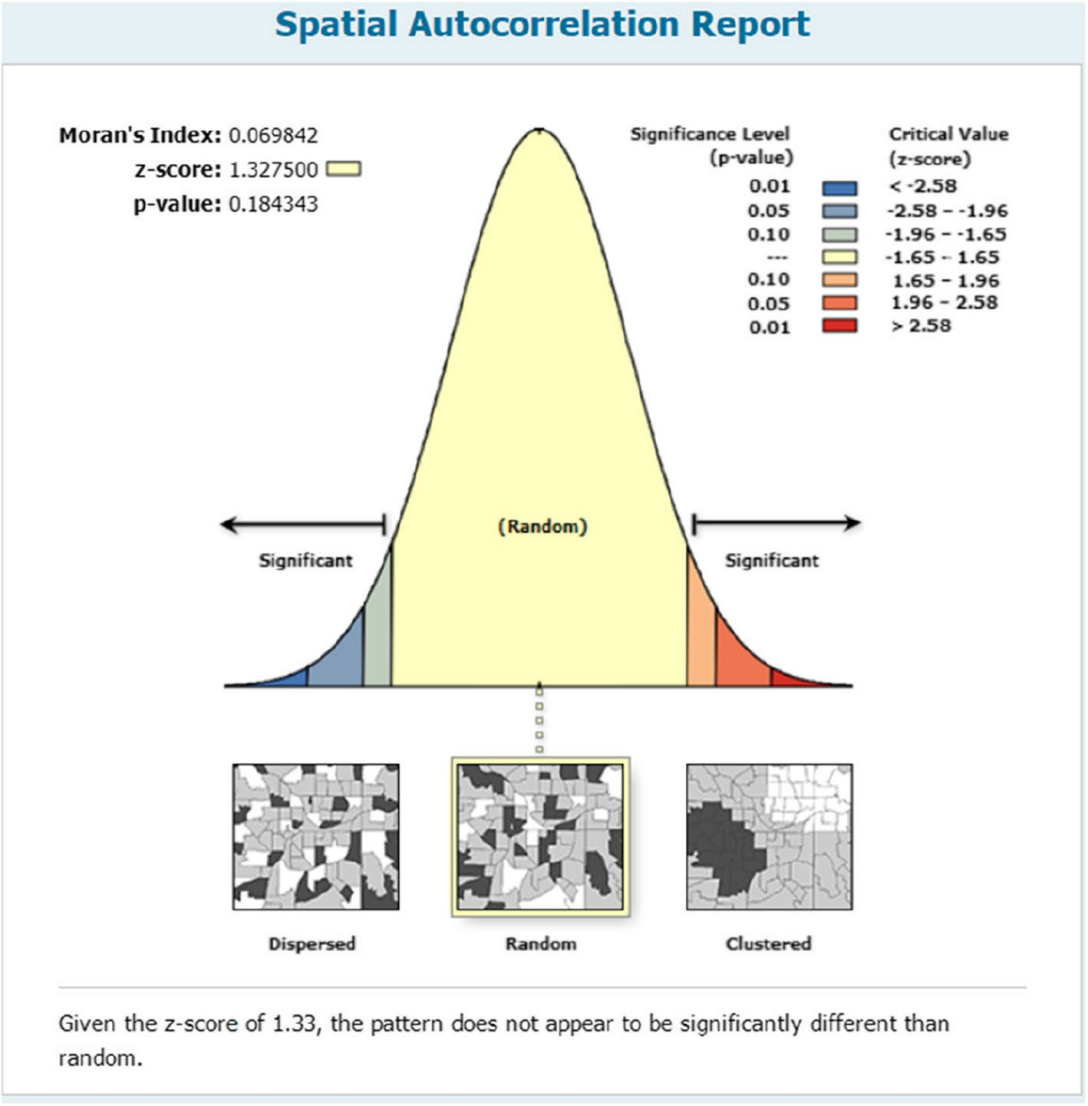
Spatial autocorrelation figure

Figure 8 below shows the spatial autocorrelation for CHC visits in 2020. There is no significance of spatial autocorrelation at the 95% confidence interval.

### Hospital visit spatial features

**Fig. 9:**
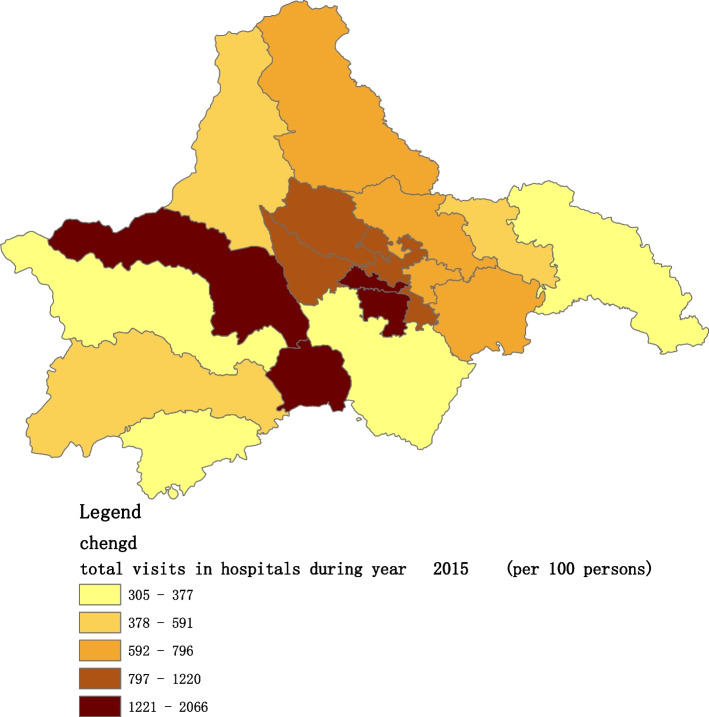
Spatial distribution of residents' visits to large hospitals in 2015

**Fig. 10:**
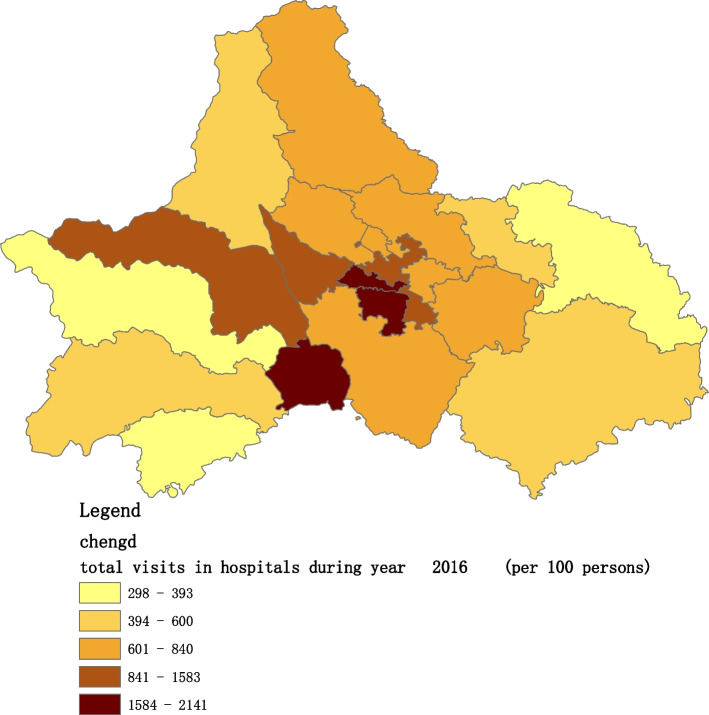
Spatial distribution of residents' visits to large hospitals in 2016

**Fig. 11:**
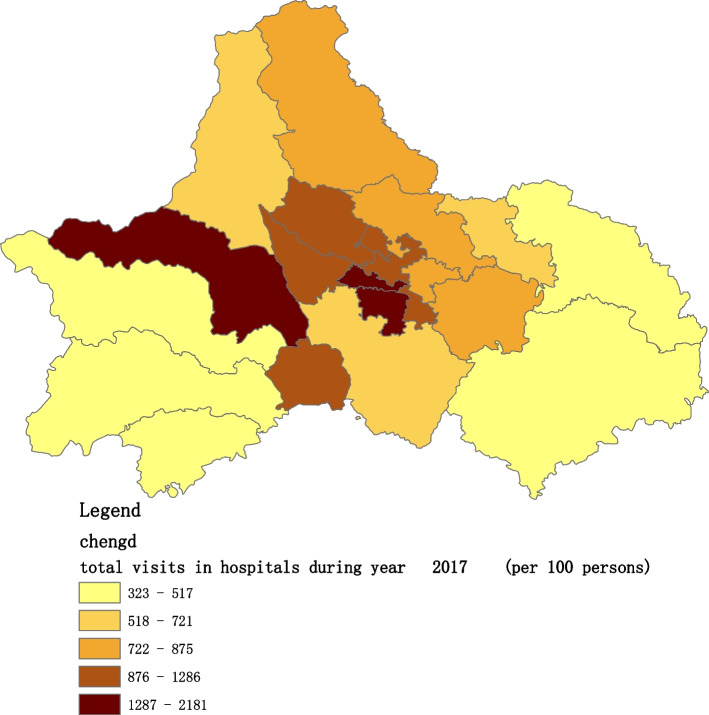
Spatial distribution of residents' visits to large hospitals in 2017

**Fig. 12:**
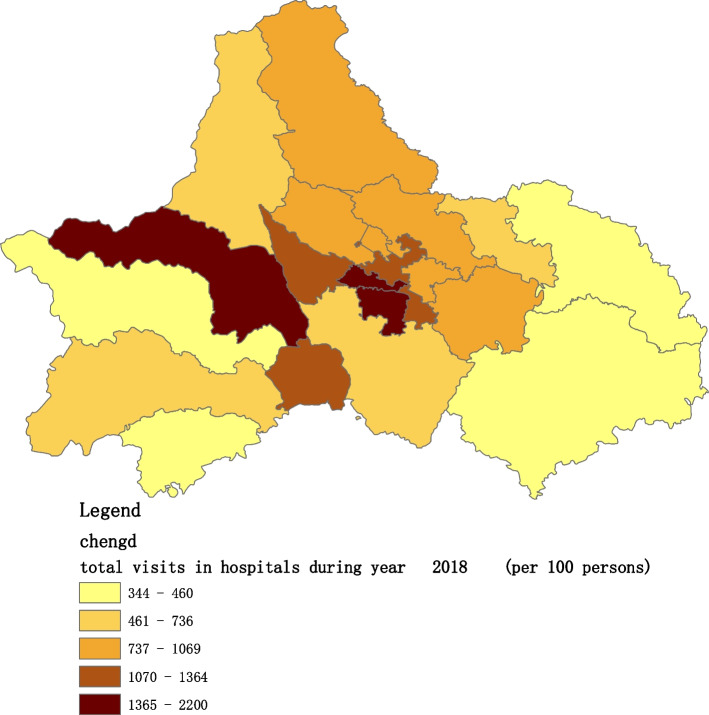
Spatial distribution of residents' visits to large hospitals in 2018

**Fig. 13:**
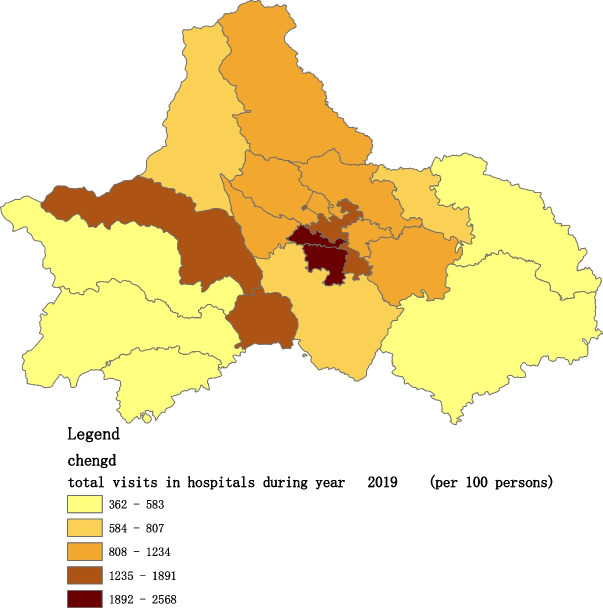
Spatial distribution of residents' visits to large hospitals in 2019

**Fig. 14:**
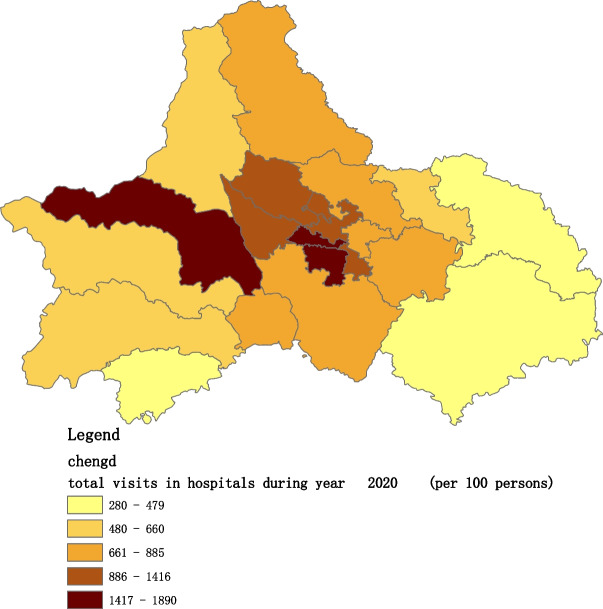
Spatial distribution of residents' visits to large hospitals in 2020

Figures 9, 10, 11, 12, 13 and 14 below show the average visit of the population to the hospital from 2015-2020. District 4 and district 2 have the most visits to the hospital.

**Fig. 15:**
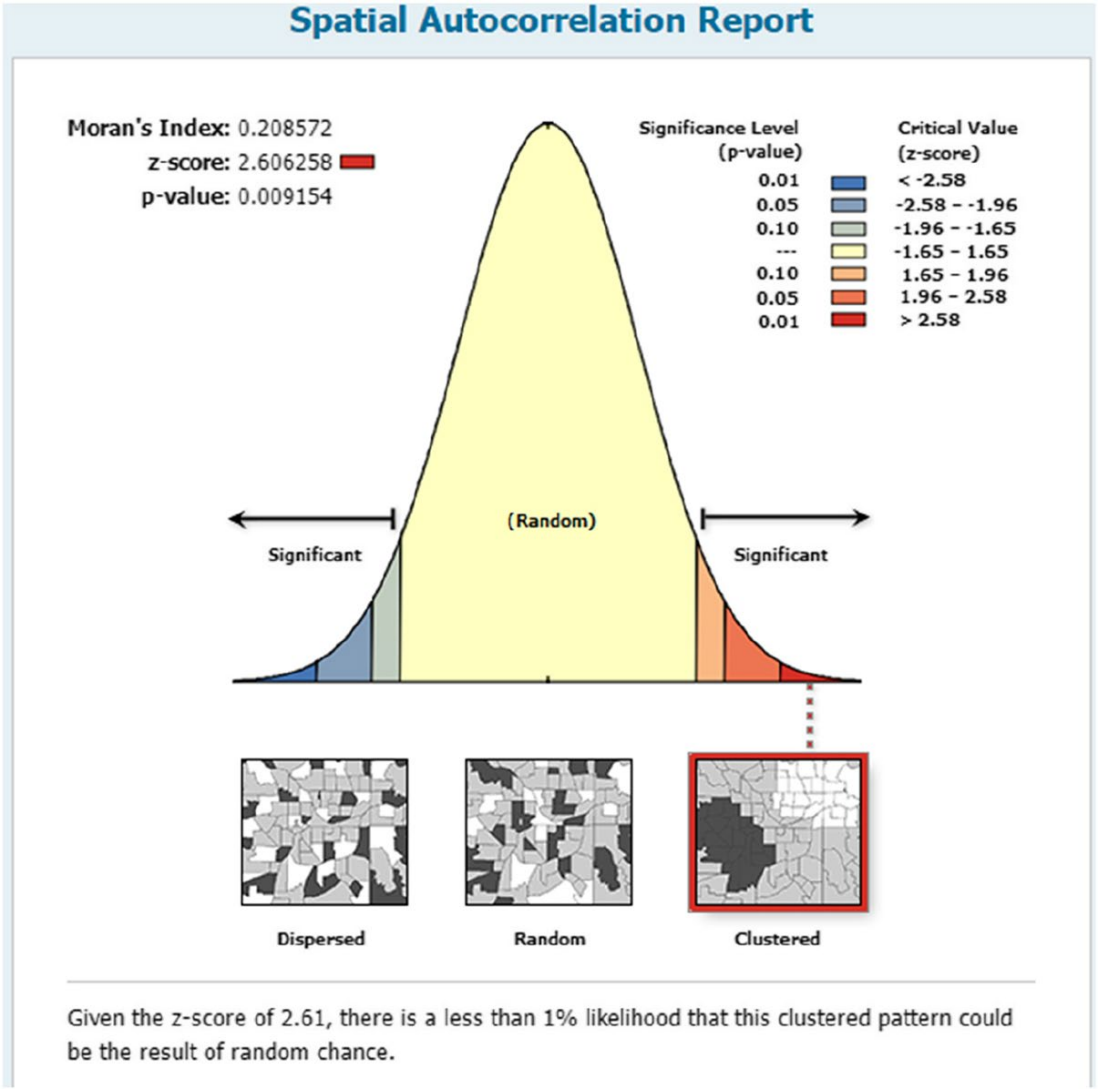
Spatial autocorrelation Fig. 2 for hospital visits

Figure 15 shows the spatial autocorrelation for hospital visits from 2020. From 2016-2020, there is significance of spatial autocorrelation at the 95% confidence interval.

**Fig. 16:**
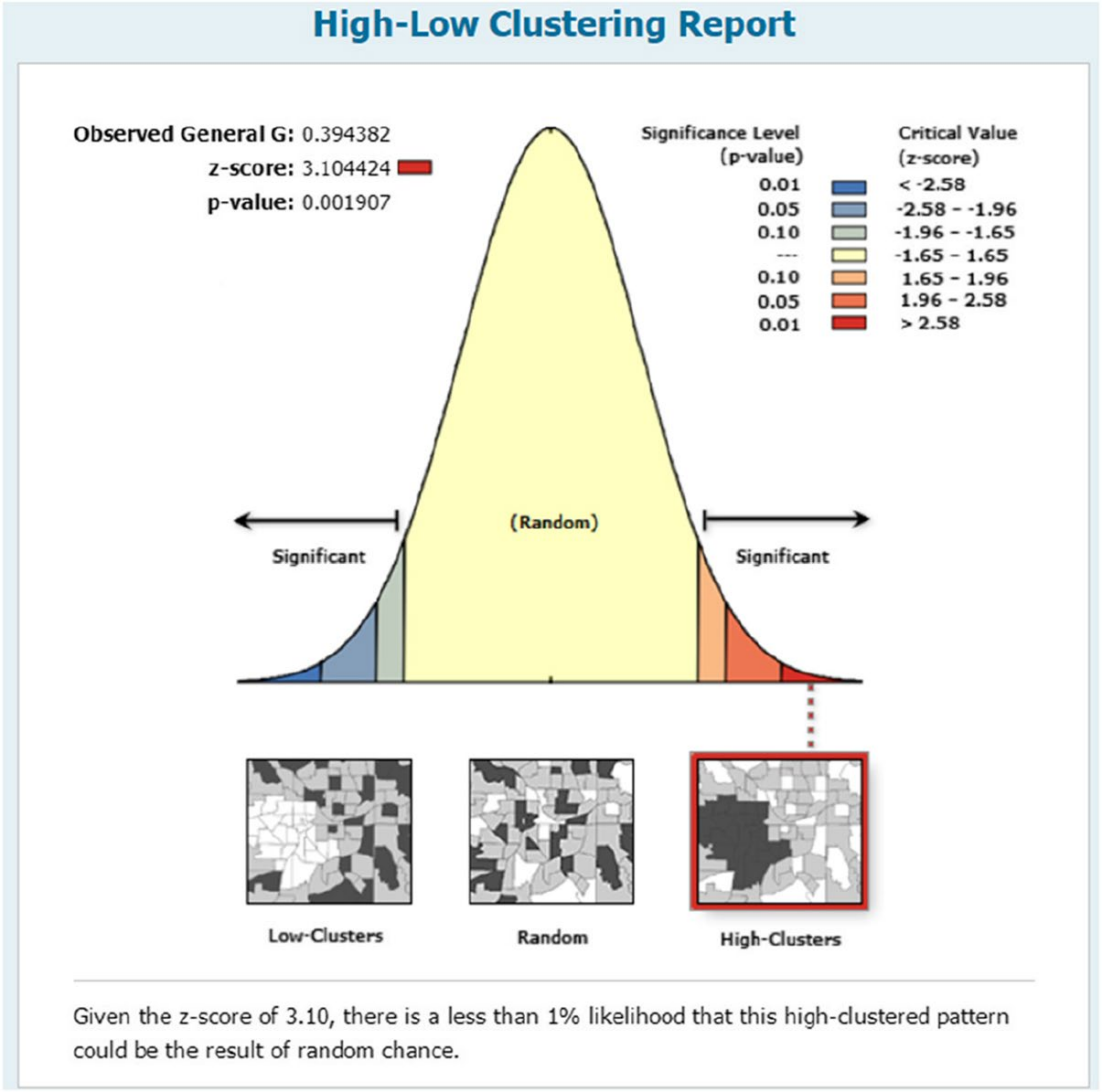
High-low cluster report of hospital visits

In Figure 16, a high-low cluster report is shown. From 2016-2020, the *p* value is below 0.05 at the 99% confidence interval, and the observed general G is above the expected general G, which means that from 2016-2020, the number of hospital visits is a high-high cluster, and the density is a spatial overflow.

### Evolution of family doctor contract service policy

During the period of 2015-2020, the family medical contract policies of 20 different administrative regions in Chengdu were sorted and analyzed. The aspects involved in each policy or specific measure were subdivided into four dimensions: leadership & governance, financing, workforce, and service delivery (Table 2 Evolution of family doctor contract service policy).

**Table 2:** Evolution of family doctor contract service policy

From YEAR 2015 to 2020	leadership &governmence	financing	workforce	service delivery
district 1	✓(YEAR 2015)	✓ (2020)	✓ (after YEAR 2020)	✓ (2016,2023)
district 2	□ (before YEAR 2015)	□ (before YEAR 2015)	✓ (after YEAR 2020)	□ (before YEAR 2015)
district 3	✓ (YEAR 2016)	□ (before YEAR 2015)	✓ (after YEAR 2020)	✓ (YEAR 2016-2018)
district 4	✓ (YEAR 2016)	□ (before YEAR 2015)	✓ (YEAR 2016)	✓ (YEAR 2016-2018)
district 5	✓ (YEAR 2018)	□ (before YEAR 2015)	✓ (YEAR 2017)	✓ (YEAR 2017)
district 6	✓ (YEAR 2020)	□ (before YEAR 2015)	✓ (YEAR 2020)	✓ (YEAR 2020)
district 7	✓ (after YEAR 2020)	□ (before YEAR 2015)	✓ (after YEAR 2020)	□ (before YEAR 2015)
district 8	✓ (YEAR 2018)	NONE	✓ (YEAR 2018)	✓ (YEAR 2018)
district 9	✓ (YEAR 2018)	□ (before YEAR 2015)	✓ (after YEAR 2020)	✓ (YEAR 2018)
district 10	✓ (YEAR 2020)	✓ (2018)	✓ (after YEAR 2020)	✓ (YEAR 2020)
district 11	✓ (YEAR 2018)	□ (before YEAR 2015)	✓ (after YEAR 2020)	✓ (YEAR 2018)
district 12	✓ (YEAR 2017)	✓ (2020)	✓ (after YEAR 2020)	✓ (YEAR 2017)
district 13	✓ (YEAR 2018)	✓ (2020)	✓ (after YEAR 2020)	None
district 14	✓ (YEAR 2019)	NONE	✓ (after YEAR 2020)	None
district 15	✓ (after YEAR 2020)	NONE	NONE	✓ (YEAR 2017)
district 16	✓ (after YEAR 2020)	NONE	✓ (after YEAR 2020)	✓ (after YEAR 2020)
district 17	✓ (after YEAR 2020)	NONE	✓ (after YEAR 2020)	✓ (after YEAR 2020)
district 18	✓ (after YEAR 2020)	NONE	✓ (after YEAR 2020)	NONE
district 19	✓ (after YEAR 2020)	✓ (YEAR 2019)	✓ (after YEAR 2020)	□ (before YEAR 2015)
district 20	✓ (after YEAR 2020)	NONE	✓ (after YEAR 2020)	NONE

Most districts had published family doctor contract service policies. Among the downtown zone, four (districts 1, 2, 3, and 4) of five (districts 1, 2, 3, 4, and 5) started relatively early approximately 2015, especially in leadership, governance, and financing. Among the second layer of the city (6,7.8,9,10,11), four of them (6,7.,9,11) started early in the family doctor contract financing arrangement, and some started the family doctor contract workforce arrangement after 2020, which means that the aspects of the policy are not coordinated. In addition, among the third layer of the city. (district 12, 13, 14, 15, 16, 17, 18, 19, 20) most family doctor contract policy started after YEAR 2020, which can be compared with other districts whether the family doctor contracting system is implemented.

## Discussion

### Spatial features

From the Administrative district chart of Chengdu city, district 1, 2, 3, 4, 5 are regarded as downtown zone, 6,7.8,9,10,11 are regarded as the second layer of the city , district 12, 13, 14, 15, 16, 17, 18, 19, 20 are regarded as the third layer of the city.

The figures show that the tendency of visits to hospitals is downtown zone > the second layer of the city > the third layer of the city and shows a high-high density tendency. However, the visits in CHCs tend to be random, and there is no autocorrelation in different districts.

The tendency of visits to hospitals reflects the medical level and residents’ preferences. The downtown zone, especially districts 2 and 4, has the most recognized large hospitals in Chengdu. The centralization tendency is significant from 2015-2020, despite any family doctor contract policy.

The high-high density implies that some medical alliances have a positive effect on promoting residents’ confidence level, and

The second layer of the city has the most benefit (West China hospital medical alliances in districts 6, 7.8, 9, 10, and 11). However, perhaps due to some traffic distance, some third layer of the city (districts 12 and 19) has little chance to visit the high-level hospital but to visit the hospital locally.

For primary care, the figure shows that there is no CHC brand that draws residents’ preferences. On the one hand, it has a positive meaning that residents choose their primary care locally. On the other hand, the whole system—the hierarchical medical system referred to by the CHCs—is not well built. In fact, to strengthen primary care, the Chinese government set up a 15-minute health care square, aiming “Minor illness does not leave the village, serious illness does not leave the county” [18]. However, patients had a strong preference for free choice between general practitioners and specialists, and the spatial feature had its own feature. There is no significance of spatial autocorrelation in 2015-2020, which means that it is randomly chosen by residents, and a high-high density trend appears in hospital visits. District4 has achieved a good demonstration role since it was officially awarded the title of the first batch of "National Chronic Disease Comprehensive Prevention and Control Demonstration Zone" in 2012. In recent years, District 4 has given full play to the government's leading role in the prevention and control of chronic diseases, striving to build a professional collaboration system for chronic disease prevention and treatment, actively promoting the national healthy lifestyle and other characteristic practices and experiences, and establishing multidepartment and street-level comprehensive prevention and control linkage mechanisms for chronic diseases. Further research revealed that grassroots hospitals may have achieved innovation and breakthroughs in policy implementation, talent introduction, diagnosis and treatment technology and innovation, driving the improvement of the overall technical level of grassroots hospitals.

Additionally, in hospital visits, West China hospital, which represents the high level in the country located in district 2 and district 4, especially in district 13, may be contributed by the Chengdu (main district)-Jianyan highway. The convenience brings more patients directly to Chengdu city, leading to spatial autocorrelation in Chengdu city.

Both hospital and CHC visits show spatial autocorrelation and high-high density. On the one hand, it is a positive reflection that both hospitals and CHCs have their own brand influence for residents; on the other hand, hospitals may still “siphon” patients around the areas, which reveals that they are not good omen to build orderly visits and hierarchical medical systems.

### Policy suggestions

Strengthen the coordination between CHC institutions and hospitals by the path of FDs

Fragmented delivery and the capacity gap between the hospital and CHC suppressed the development of primary care. The family doctor contract reveals an ideal path to accomplish the above goal.

hospital-centered fragmented delivery [19] were to prevail—population health outcomes would suffer; health-care expenditures would escalate, with patients bearing increasing costs; and a two-tiered system would emerge in which access and quality of care are decided by ability to pay. We then propose an alternative pathway that includes the reform of public hospitals to pursue the public interest and be more accountable, with public hospitals as the benchmarks against which private hospitals would have to compete, with performance-based purchasing, and with population-based capitation payment to catalyze coordinated care. Any decision to further expand the for-profit private hospital market should not be made without objective assessment of its effect on China's health-policy goals.

To establish a medical alliance or integrated delivery system, as encouraged by the State Council of China and recommended globally, CHC institutions and hospitals need to closely coordinate their functions. In addition to vertical technical support provided by hospitals to CHC institutions within the same catchment areas, deeper coordination between them should be implemented to best suit local contexts, with integrated systems for staff training, medication supply, and health information technology support.

However, the most significant issue is to draw the incentive during the collaboration among hospitals to the CHC. CHC providers are in a central position to coordinate a person's care needs, from prevention to disease management to curative care. There are several barriers to overcome before this aspiration becomes a reality. The concept of continuity of care entails several dimensions. First, relational continuity encourages patients to enter into contractual arrangements with family doctors. However, China does not make it compulsory for patients to see CHC providers as their first contact. As the first step toward building a gatekeeping system, the government has introduced a family doctor registration policy by which each resident would be registered with a team of family doctors. Moreover, there is a general lack of patient awareness about the importance of continuity of care. In a study in Beijing, patients had a strong preference for free choice between general practitioners and specialists. Thus, there is an essential way to build FDs where CHC institutions and hospitals participate together. By the path of FDs, CHC institutions and hospitals both sign contrast with the residents and take responsibility for the residents.

Reform the payment by integrating CHC institutions and hospitals by the path of FDs

In 2015, the Chinese government issued guidelines for building a so-called tiered health-care delivery system whereby each level of health-care facility (tertiary, secondary, and primary) would deliver care according to their designated functions; care across the levels was to be integrated and coordinated with bidirectional referral mechanisms through establishing medical alliance or integrated systems. These pilot implementations have been slow and hindered by several factors. First, as hospitals and CHC institutions are still primarily paid by fee-for-service, they compete for patients and have few incentives to coordinate. Second, the social health insurance programme, which covers 96% of the population, 4 reimburses patients wherever they seek care without referral; thus, there is generally no defined coordinating process. In addition, reimbursement for hospital care is more generous than that for care at CHC institutions considering the ceiling and therefore encourages patients to bypass CHC facilities, making it difficult for CHC providers to function as gatekeepers. Third, electronic patient records are not integrated and are seldom shared between CHC institutions and hospitals. 56 Therefore, even though partnerships between hospitals and CHC institutions are encouraged and have formed in many cities, 57 the association remains loose.

Within CHC institutions, the National Basic Public Health Service Program could in theory provide a basis for integration between clinical care and public health services. However, the integration was suboptimal in reality for two reasons. First, financing for public health services and clinical care of the same CHC institutions came from different sources. Although the government directly funds a defined package of public health services, clinical care is funded by social health insurance. Second, there is almost no coordination in monitoring, performance measurement, or management between the two programs. Thus, as we observed as researchers and practitioners, there is little workflow interaction or information sharing between the programs. For instance, in hypertension management visits under the National Basic Public Health Service Program, patients can have blood pressure measurements and lifestyle consultations by public health workers but cannot obtain prescriptions of antihypertensive drugs without attending clinics. Additionally, resident health records of public health services and medical records of clinical care are kept by two separate information systems even for the same visit of the same patient, without linkage between them. Poor care coordination is a hindrance, particularly to managing noncommunicable diseases. institutions, the opportunity to integrate clinical care and public health services is severely limited. Thus, by providing the FDs that the CHC institutions and hospitals both involved, the payment should be integrated.

Either public or private packages could be provided to meet the devise health needs of citizens.

Enhance the quality of training for the new and current CHC workforce in the path of FDs

The State Council issued guidance on the reform and development of training and incentive mechanisms for CHC physicians. Despite this guidance, a comprehensive range of detailed recommendations on the quality of training are needed to address the wide variation in standards of medical school education [20, 21]. Without the family doctor contract mode, the concept of patient-centered care is difficult to teach during college training and GP resident training periods. The curriculum needs to be promoted without teaching GP residents to take responsibility for FDs.

In CHC staff training, it is necessary to tailor the clinical practice guidelines to the CHC environment and contain feasible and affordable suggestions, including the integration of patient-centered views with patient goals [22, 23]. These guidelines should focus on the use of cost-effective diagnostic methods and treatment measures [24–27]. China will benefit from an institution that supervises the formulation of disease management protocols and involves CHC providers, which in turn can provide information for the training of CHC doctors in appropriate and contextual use. FDs provide GPs and specialist training with a common goal for person-centered care.

Integrate in referral system in tern of path of FDs

The role of family physicians in referring patients to specialists diminishes, possibly because the current referral system is imperfect [28]. One study criticized two-way referrals for transferring patients only from community health centers to hospitals but not from hospitals to community health centers. There has not been enough focus on increasing the attractiveness of community health centers, and there is an urgent need for a convenient and effective referral system [23, 29, 30]. It is also necessary to increase the motivation of secondary and tertiary hospitals to admit patients from community health centers. Surprisingly, we did not find a positive effect of first exposure to CHC on referral through CHC. In other words, although contracting with FD patients was positively associated with referral behavior, it was not associated with first contact with CHC patients. It is worth exploring how functional development affects recommendation behavior in further research. However, other researchers have reported similar results. Recent research suggests that gatekeeping may not be associated with changes in the coordination of referral care, although it is associated with a wider range of conditions managed by GPs at first contact. In addition, an inverse association was found, suggesting that CHC gatekeeping can reduce hospitalizations. Although we found that FDs have a positive effect on first contact and referral through CHC, the mechanisms by which FDs affect referral behavior remain unclear. The cooperation of the feature development team may bring about positive and negative mechanisms that influence recommendation behavior.

## Conclusion

China has made much effort to build a hierarchical medical system and implement a family doctor contract policy to speed up this goal. However, the resident choice is currently free, and the spatial features reveal some reality regarding how the residents choose to visit the doctor. This study provides spatial features for visits in primary health care and hospitals to draw implications for family doctor contracts.

## Availability of data and materials

All data and materials are available. The datasets generated for this study are available by contacting the corresponding author.

### Supplementary Information

**Additional file 1: Appendix.** Administrative district of Chengdu city.

## Data Availability

All data and materials are available. The datasets generated for this study are available by contacting the corresponding author. All the administrative zoning map is from China. Map No. GS(2020)3022 approved by the Ministry of Natural Resources and the administrative zoning map of city of Cheng is attached in Appendix.

## Abbreviations

GP: Is an abbreviation for general practice
CHC: Is an abbreviation for community health center
FDs: Is an abbreviation for family doctor contract service
