# Supplementary material for: A spatial feature analysis of primary health care utilization in a large city in China and its implications for family doctor contract service policy
Source: BMC Health Serv Res. 2024 Feb 13;24:194. doi: 10.1186/s12913-023-10389-8 (PMC10863294; doi:10.1186/s12913-023-10389-8)
Supplement: Supplementary file 1 — Additional file 1: Appendix. Administrative district of Chengdu city. [file 12913_2023_10389_MOESM1_ESM.docx]

APPENDIX

Administrative district of Chengdu city
